# Vertical organic electrochemical transistors for complementary circuits

**DOI:** 10.1038/s41586-022-05592-2

**Published:** 2023-01-18

**Authors:** Wei Huang, Jianhua Chen, Yao Yao, Ding Zheng, Xudong Ji, Liang-Wen Feng, David Moore, Nicholas R. Glavin, Miao Xie, Yao Chen, Robert M. Pankow, Abhijith Surendran, Zhi Wang, Yu Xia, Libing Bai, Jonathan Rivnay, Jianfeng Ping, Xugang Guo, Yuhua Cheng, Tobin J. Marks, Antonio Facchetti

**Affiliations:** 1 School of Automation Engineering, University of Electronic Science and Technology of China (UESTC), Chengdu, China; 2 Department of Chemistry and the Materials Research Center, Northwestern University, Evanston, IL USA; 3 Department of Chemical Science and Technology, Yunnan University, Kunming, China; 4 Department of Materials Science and Engineering and the Shenzhen Key Laboratory for Printed Organic Electronics, Southern University of Science and Technology (SUSTech), Shenzhen, China; 5 School of Biosystems Engineering and Food Science, Zhejiang University, Hangzhou, China; 6 Innovation Platform of Micro/Nano Technology for Biosensing, ZJU-Hangzhou Global Scientific and Technological Innovation Center, Hangzhou, China; 7 Department of Biomedical Engineering, Northwestern University, Evanston, IL USA; 8 College of Chemistry, Sichuan University, Chengdu, China; 9 Air Force Research Laboratory, Materials and Manufacturing DirectorateWPAFB, Ohio, OH USA; 10 School of Materials Science and Engineering, North University of China, Taiyuan, China; 11 Flexterra Inc. 8025 Lamon Avenue, Skokie, IL USA

**Keywords:** Electronic devices, Electrical and electronic engineering, Electrochemistry, Electronics, photonics and device physics

## Abstract

Organic electrochemical transistors (OECTs) and OECT-based circuitry offer great potential in bioelectronics, wearable electronics and artificial neuromorphic electronics because of their exceptionally low driving voltages (<1 V), low power consumption (<1 µW), high transconductances (>10 mS) and biocompatibility^1–5^. However, the successful realization of critical complementary logic OECTs is currently limited by temporal and/or operational instability, slow redox processes and/or switching, incompatibility with high-density monolithic integration and inferior n-type OECT performance^6–8^. Here we demonstrate p- and n-type vertical OECTs with balanced and ultra-high performance by blending redox-active semiconducting polymers with a redox-inactive photocurable and/or photopatternable polymer to form an ion-permeable semiconducting channel, implemented in a simple, scalable vertical architecture that has a dense, impermeable top contact. Footprint current densities exceeding 1 kA cm^−2^ at less than ±0.7 V, transconductances of 0.2–0.4 S, short transient times of less than 1 ms and ultra-stable switching (>50,000 cycles) are achieved in, to our knowledge, the first vertically stacked complementary vertical OECT logic circuits. This architecture opens many possibilities for fundamental studies of organic semiconductor redox chemistry and physics in nanoscopically confined spaces, without macroscopic electrolyte contact, as well as wearable and implantable device applications.

## Abstract (web summary)

Vertical organic electrochemical transistors demonstrating unprecedented performances in both p- and n-type operation modes have been synthesized from new electro-active and ion-permeable semiconducting polymers by the interface engineering of electro-active blend layers.

## Main

Organic electrochemical transistors (OECTs) are attractive for bioelectronics, wearable electronics and neuromorphic electronics because of their low driving voltage, low power consumption, high transconductance and facile integration in mechanically flexible platforms^1–3,5,9–11^. However, further OECT advances face challenges. (1) Despite progress^8^, poor electron-transporting (n-type) OECT performance versus their hole-transporting (p-type) counterparts (approximately 1,000 times lower transconductance and/or current density)^6,7,12^, hinders the development of complementary logic and sensitivity to in vivo relevant analyte cations (for example, Na^+^, K^+^, Ca^2+^, Fe^3+^ and Zn^2+^) for biosensor development. (2) Temporal and/or operational instability hinders all possible applications. (3) Unbalanced p-type and n-type OECT performance prevents integration into complementary circuits^13,14^. (4) Slow redox processes lead to sluggish switching. (5) State-of-the-art conventional OECTs (cOECTs), having planar source–drain electrode architectures, require small channel lengths (*L*) of at most 10 µm, along with precisely patterned semiconducting layers and electrode coatings with passive materials, for high transconductance (*g*_m_) and fast switching (approximately in the millisecond range)^15^, requiring complex fabrication methodologies^15,16^. Note that conventional photolithography can only reliably realize features or *L* larger than 1 µm (ref. ^16^), and although printing and laser cutting offer simplified cOECT fabrication, this is at the expense of performance^17–19^. Moreover, to increase *g*_m_, OECTs typically use thick semiconducting films, inevitably compromising switching speeds because high *g*_m_ values require efficient ion exchange between the electrolyte and the bulk semiconductor^20^. Consequently, without progress in materials design, particularly for n-type semiconductors, and the realization of new device architectures, OECT applications will remain limited in scope.

**Fig. 1:**
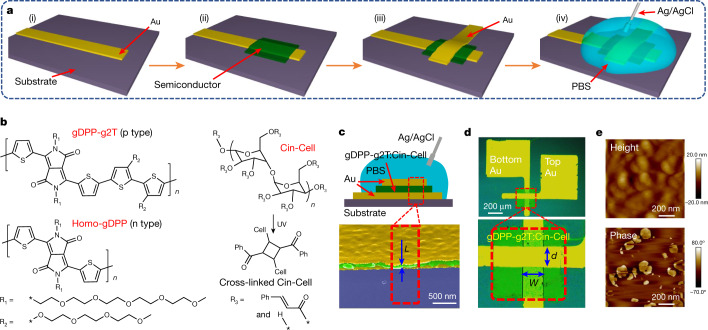
Fabrication scheme and vOECT materials used. **a**, Fabrication process for vOECTs: thermal evaporation of the bottom source electrode with a shadow mask (i), spin-coating and photopatterning of the semiconducting polymer + Cin-Cell blend (ii), thermal evaporation of the top drain electrode with a shadow mask (iii) and application of phosphate buffer solution (PBS) electrolyte and Ag/AgCl gate electrode (iv). **b**, Chemical structures of the redox-active semiconducting polymers (gDPP-g2T (p-type); Homo-gDPP (n-type)), and the redox-inactive cross-linkable polymer (Cin-Cell, cross-linking occurs through a photo-induced 2 + 2 cycloaddition reaction). **c**, Cross-section illustration of p-type vOECTs, along with a false-coloured cross-section SEM image showing the phase-separated layer sandwiched between two dense Au electrodes. **d**, Optical image of a p-type vOECT, in which the electrode overlapping area is enlarged (*W* = *L* = 70 μm). **e**, AFM height and phase images of gDPP-g2T:Cin-Cell blends. In all samples, the gDPP-g2T:Cin-Cell weight ratio is 9:2.

**Extended Data Fig. 1:**
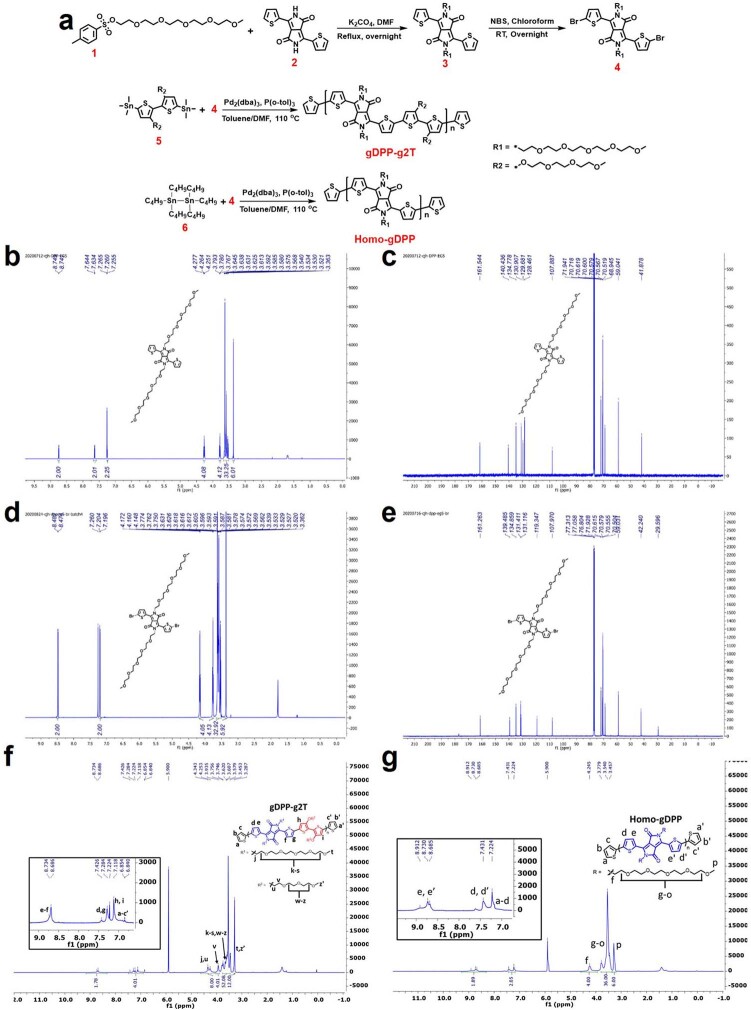
Synthesis and characterizations of gDPP-g2T and Homo-gDPP. **a**, Synthetic route to the semiconductors gDPP-g2T (p-type) and Homo-gDPP (n-type). **b**, ^1^H NMR spectrum of compound **3**. **c**, ^13^C NMR spectrum of compound **3**. **d**, ^1^H NMR spectrum of compound **4**. **e**, ^13^C NMR spectrum of compound **4**. **f**, ^1^H NMR spectrum of polymer gDPP-g2T. **g**, ^1^H NMR spectrum of polymer Homo-gDPP. Source data

**Extended Data Fig. 2:**
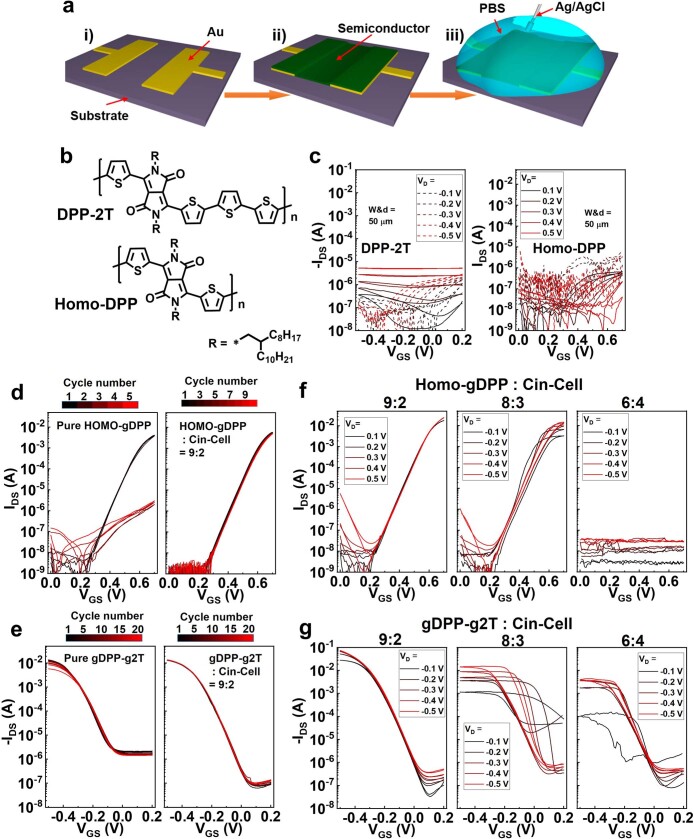
Fabrication process for conventional planar OECTs, performance evaluation for vOECTs based on polymers DPP-2T, Homo-DPP, and vOECTs with and without different Cin-Cell contents in the active layer. **a**, Fabrication process for a conventional OECT (cOECT). i) Source/drain Au electrode fabrication (this involves photolithography, Au thermal evaporation, and lift-off processes); ii) spin-coating of the semiconducting layer and UV cross-linking; iii) PBS and Ag/AgCl as electrolyte dielectric and gate electrode. **b**, Chemical structures of the redox-inactive semiconducting polymers **DPP-2T** (p-type); **Homo-DPP** (n-type). **c**, Representative transfer characteristics of p-type **DPP-2T** and n-type **Homo-gDPP** based vOECTs. **d**, Representative transfer characteristics of (**d**) n-type vOECTs with a pristine **Homo-gDPP** and **Homo-gDPP:Cin-Cell** (9:2) as the semiconductor channel and (**e**) p-type vOECTs with a pristine **gDPP-g2T** and **gDPP-g2T:Cin-Cell** (9:2) as the semiconductor channel. W= d = 30 µm, V_D_ = ±0.1 V. Representative transfer characteristics of (**f**) **Homo-gDPP:Cin-Cell** and (**g**) **gDPP-g2T:Cin-Cell** based vOECTs with weight ratios of 9:2, 8:3 and 6:4, respectively. W= d = 50 µm. Source data

In this report, we demonstrate high-performance p- and n-type OECTs and complementary circuits by using a vertical device architecture (vertical OECT, hereafter named vOECT) readily fabricated by thermal evaporation and masking of impermeable and dense Au source–drain electrodes and spin-coating and photopatterning of an ion-conducting semiconductor channel. The vOECT fabrication process is illustrated in Fig. 1a and details can be found in the Methods. The key to this process is the use of a redox-active p-type (gDPP-g2T) or n-type (Homo-gDPP) semiconducting polymer blended with a redox-inert and photocurable polymer component (cinnamate-cellulose polymer (Cin-Cell)) as the OECT channel (see the structures in Fig. 1b, the synthesis process in the Methods and Extended Data Fig. 1). On the basis of the control experiments (vide infra) the optimal semiconducting polymer:Cin-Cell weight ratio was found to be 9:2. A vOECT geometry cross-section and selected optical and scanning electron microscopy (SEM) images (Fig. 1c,d) indicate that the channel length (*L*) is the semiconductor layer thickness (approximately 100 nm), the widths of the bottom and the top electrodes define the channel width (*W*) and the nominal depth (*d*) of the semiconductor, respectively. cOECTs and vOECTs that use polymers without ion-conducting ethylene glycol side chains were also fabricated as controls; their performance is marginal (Extended Data Fig. 2).

**Extended Data Fig. 3:**
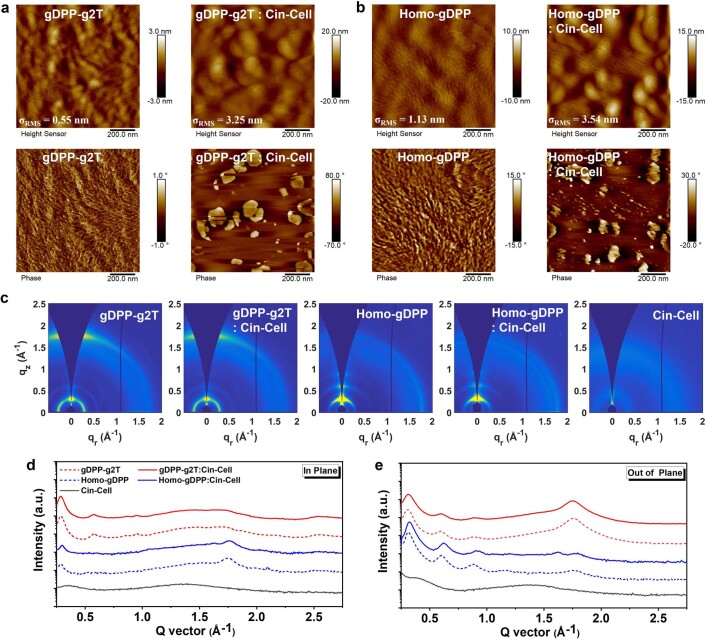
Semiconductor film morphologies and microstructures. **a**, AFM height images and phase images of a pristine **gDPP-g2T** film and **gDPP-g2T:Cin-Cell blend** (9:2 in mass) film. **b**, AFM height images and phase images of a pristine **Homo-gDPP** film and a **Homo-gDPP:Cin-Cell** blend (9:2 in mass) film. **c**, 2D-GIWAXS images and corresponding (**d**) in-plane and (**e**) out-of-plane one-dimensional line-cuts for **gDPP-g2T**, **gDPP-g2T**: **Cin-Cell**, **Homo-gDPP**, **Homo-gDPP**: **Cin-Cell**, and **Cin-Cell** films. The **gDPP-g2T** in both pristine and blend films exhibits a preferential π-face-on orientation, yet with a considerable portion of crystallites being edge-on oriented. In all cases the lamellar and π-π periodicities are pinned at 20.2 Å and 3.6 Å, respectively. However, in both **Homo-gDPP** and **Homo-gDPP:Cin-Cell** the polymer orientation is preferentially edge-on and while the π-π distance is pinned at 3.6 Å, and the lamellar spacings vary slightly, from 20.2 Å to 19.6 Å, respectively. Source data

Before evaluation of the device, the morphology and microstructure of the semiconducting polymer:Cin-Cell blend were characterized. As shown in Extended Data Fig. 3a,b, the pristine gDPP-g2T and HOMO-gDPP films are continuous and smooth (root-mean-square roughness, *σ*_r.m.s._ ≈ 1 nm), whereas both polymer blends with Cin-Cell are rougher after ultraviolet (UV) cross-linking/patterning (*σ*_r.m.s._ ≈ 3 nm) with evidence of phase separation in atomic force microscopy (AFM) (Fig. 1e), in which the Cin-Cell forms pillar-like structures that should enhance the structural robustness and stability. Thus, the Cin-Cell in the semiconducting matrix acts not only as a photopatterning component of the channel, but, most importantly, as an OECT structural stabilizer (vide infra). The two-dimensional grazing incidence wide-angle X-ray scattering (2D–GIWAXS, Extended Data Fig. 3c–e) patterns of the pure polymer and polymer:Cin-Cell mixture films are similar, corroborating phase separation and demonstrating that Cin-Cell addition does not substantially alter the overall film texturing and the polymer chain order.

Next, the vOECTs and cOECTs were tested and the performance parameters were extracted following standard procedures (Extended Data Table 1)^7,21^. Before discussing the results, note that control vOECTs based on semiconducting polymers without ethylene glycol side chains exhibit a negligible transistor response, demonstrating that the hydrophilic ion-complexing polymer facilitates ion penetration across the nanoscopic thin electrolyte-blend interface (Extended Data Fig. 2b,c). Furthermore, the performance of control vOECTs with varying Cin-Cell weight contents indicate (Extended Data Fig. 2d–g) that the device yield without Cin-Cell is low and, most importantly, that such devices are unstable after very few repeated gate voltage (*V*_G_) cycles, mainly reflecting top electrode delamination, whereas those that use a semiconducting polymer:Cin-Cell weight ratio of more than 9:2 exhibit poor performance (low current on (*I*_ON_) and large hysteresis), because of reduced redox-active polymer content and constricted ion diffusion. Consequently, all of the data reported here are for cross-linked semiconducting polymer:Cin-Cell blends with a 9:2 weight ratio.

**Fig. 2:**
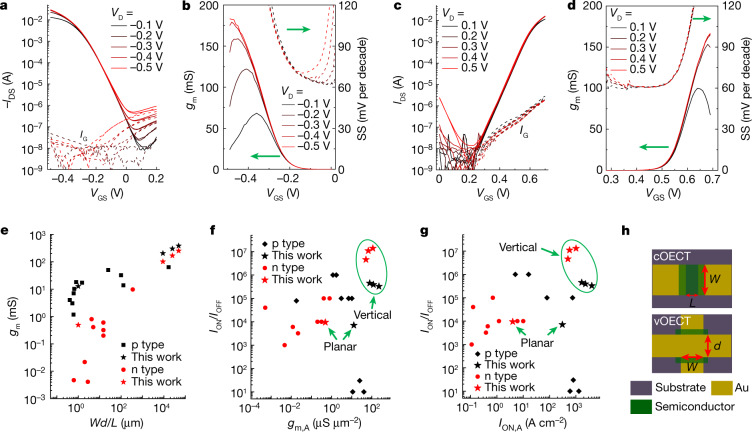
OECT performance and comparison with literature data. **a**–**d**, Representative transfer characteristics (**a**,**c**) and corresponding *g*_m_ and SS curves (**b**,**d**) of p-type gDPP-g2T (*W* = *d* = 30 µm) (**a**,**b**) and n-type Homo-gDPP (*W* = *d* = 50 µm) (**c**,**d**) vOECTs. (*L* ≈ 100 nm). **e**, *g*_m_ as a function of *Wd/L* for the present vOECTs and cOECTs as well as the previously reported OECTs^7,8,14,15,21,23–32^. Comparisons of current on/off ratio (*I*_ON_/*I*_OFF_) versus *g*_m_ per unit area (*g*_m,A_) (**f**) and on-current per unit area (*I*_ON,A_) (**g**) for different v- and cOECTs. Note, different asterisks are data of this work based on different *W* and *d* (Extended Data Table 1). **h**, Cartoon illustrating how the *g*_m,A_ and *I*_ON,A_ are calculated, where *g*_m,A _= *g*_m_/(*WL*), *I*_ON,A _= *I*_ON_*/(WL*) for cOECT, whereas *g*_m,A _= *g*_m_/(*Wd*), *I*_ON,A _= *I*_ON_/(*Wd*) for vOECT. Source data

**Extended Data Fig. 4:**
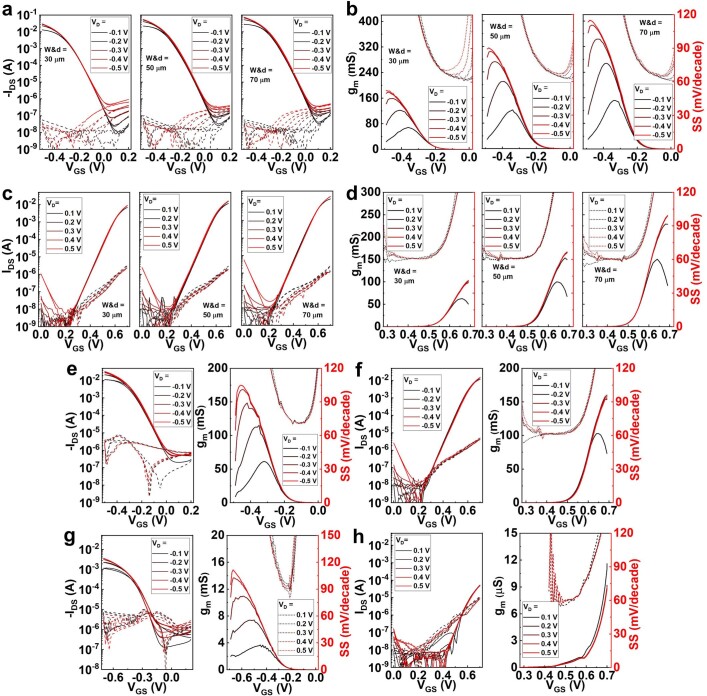
OECT performance. (**a**,**c**) Representative transfer characteristics and (**b**,**d**) corresponding g_m_ and SS curves of (**a**,**b**) p-type **gDPP-g2T:Cin-Cell** and (**c**,**d**) n-type **Homo-gDPP:Cin-Cell** vOECTs with the indicated W and d values ranging from 30, 50, and 70 µm. vOECT performance with unpatterned semiconducting layers, where **e** and **f** are representative transfer characteristics and the corresponding g_m_ and SS curves of an unpatterned p-type **gDPP-g2T:Cin-Cell** vOECT (W= d = 30 μm) and an unpatterned n-type **Homo-gDPP:Cin-Cell** vOECT (W= d = 50 μm), respectively. Impressively, these devices exhibit almost identical performance parameters (I_ON_, V_ON_, g_m_) versus those of the corresponding p- and n-type vOECTs having a patterned channel, with the exception of a higher off-current for the p-type vOECT. This is due to confinement of the charge transport within the overlapping source/drain electrodes, and the ultra-small L prevents substantial fringing effects. Representative transfer characteristics and corresponding g_m_ and SS curves for (**g**) p-type **gDPP-g2T:Cin-Cell** cOECTs and (**h**) n-type **Homo-gDPP:Cin-Cell**. Note, W = 100 µm, L = 10 µm. Source data

**Extended Data Fig. 5:**
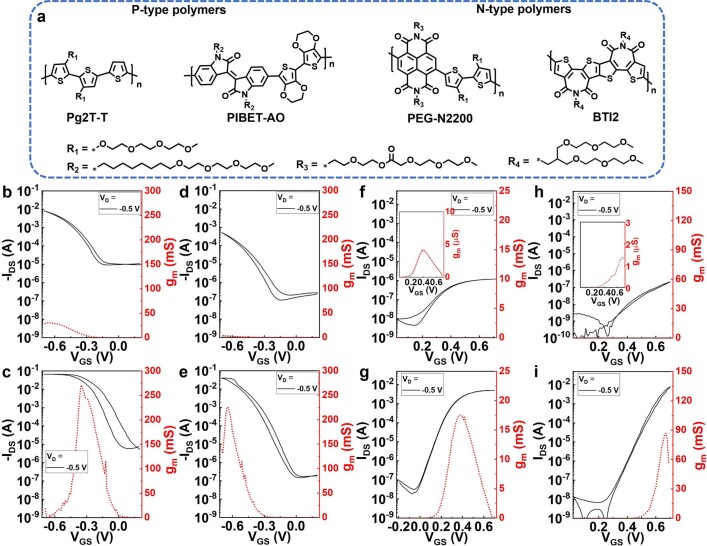
Chemical structures and OECT performance of the indicated polymer semiconductors blended with Cin-Cell. **a**, Chemical structures of the redox-active semiconducting polymers [Pg2T-T and PIBET-AO (p-type); PEG-N2200 and BTI2 (n-type)]. Representative transfer and g_m_ characteristics of planar **(b**, **d**, **f**, **and h**) and vertical (**c**, **e**, **g**, **and i**) OECTs based on Pg2T-T (**b**,**c**), PIBET-AO (**d**,**e**), PEG-N2200 (**f**,**g**), and BTI2 (**h**,**i**). Note, W and d are both 30 μm for the vOECT, while W and L are 100 µm and 10 µm, respectively, for the cOECT. For PEG-N2200 and BTI2 based planar OECTs, the g_m_s are too small when plotted on the same scale as that of the vOECT counterparts, so therefore insets with magnified Y axis are included. In all samples, the semiconductor:Cin-Cell weight ratio is 9:2. Source data

The vOECT and cOECT transfer characteristics and the corresponding *g*_m_–subthreshold swing (SS) plots (Fig. 2a–d and Extended Data Fig. 4) demonstrate extraordinary performances for both p- and n-type vOECTs, achieving maximum drain currents (*I*_ON_) of (8.2 ± 0.5) × 10^−2 ^A (drain voltage (*V*_D_) = −0.5 V, *V*_G_ = −0.5 V) and (2.5 ± 0.1) × 10^−2 ^A (*V*_D_ = +0.5 V, *V*_G_ = +0.7 V), and *g*_m_ values as high as 384.1 ± 17.8 mS and 251.2 ± 7.6 mS, respectively (Fig. 2e). Note that despite the ultra-small channel lengths (*L* ≈ 100 nm), the *I*_ON_/current off (*I*_OFF_) ratios of both devices are impressive (≥10^6^), exclusively due to the higher *I*_ON_ and low *I*_OFF_. All of the p- and n-type vOECTs retain stable turn-on voltages (*V*_ON_) of +0.10 and +0.21 V as well as SS values of approximately 60 and approximately 62 mV per decade, respectively, upon scanning *V*_D_ from ±0.1 to ±0.5 V. More relevant parameters for vertical architectures are the area-normalized *g*_m_ (*g*_m,A_) and *I*_ON_ (*I*_ON,A_) metrics^22^. As shown in Fig. 2f,g, *g*_m,A_ (*I*_ON,A_) values as high as 226.1 µS µm^−^^2^ (4,036 A cm^−^^2^) and 112.4 µS µm^−^^2^ (1,015 A cm^−^^2^) are achieved for p- and n-type vOECTs, respectively. These values are about 18 times (13 times) and 100 times (1,000 times) greater than those measured in the corresponding p- and n-type cOECTs, respectively (Extended Data Table 1). Thus, the present p-type vOECTs exhibit, to our knowledge, the highest *g*_m,A_ and *I*_ON,A_ values reported up until now, even surpassing those of heavily doped and/or depletion-mode poly(3,4-ethylenedioxythiophene):polystyrene sulfonate (PEDOT:PSS) cOECTs. In addition, to our knowledge, the present n-type vOECT performance surpasses all previously reported OECTs (including p-type OECTs) in terms of *g*_m,A_ and *I*_ON_/*I*_OFF_(refs. ^7,8,14,15,21,23–32^). Importantly, the present vOECT structures also have reduced footprints because the contacting lines also function as source and drain contacts, eliminating the need for extra source–drain pads overlapping with the channel materials that are required in cOECTs. This vertical architecture and blending strategy with the Cin-Cell is also applicable to other mixed ionic-electronic semiconductors, with the vOECTs based on two p-type (Pg2T-T and PIBET-AO) and two n-type (polyethylene glycol-N2200 (PEG-N2200) and BTI2) polymers exhibiting similarly enhanced transistor performance versus their planar counterparts (Extended Data Fig. 5).

**Extended Data Fig. 6:**
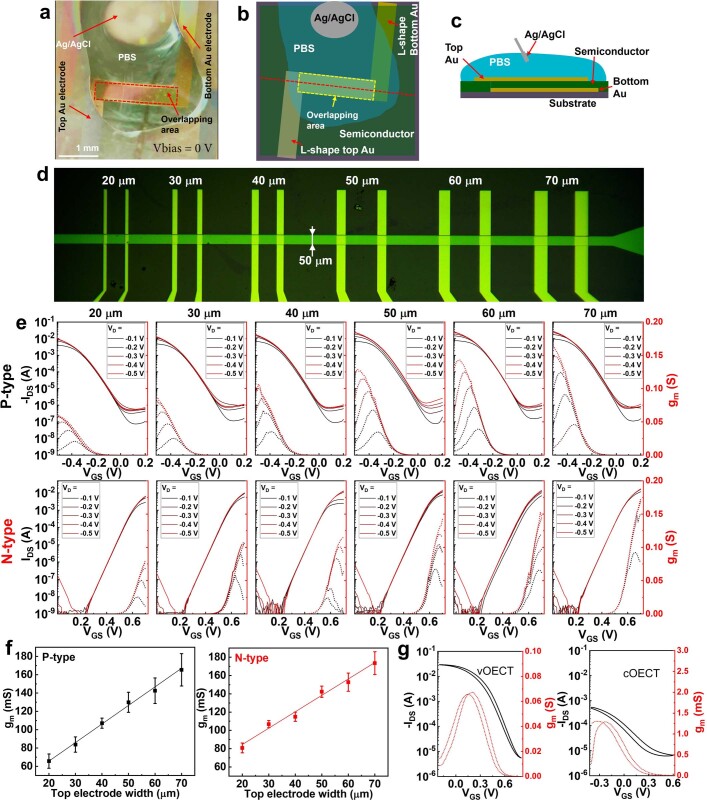
Vertical device structures for the direct observation of the electrochromic and performance characteristics of vOECTs with different top electrode widths. **a**, Top view photograph and (**b**) corresponding p-type vOECT schematic indicating the large electrode overlapping area (~2 × 0.5 mm) used to monitor the electrochromic process associated to the redox chemistry of semiconducting layer. Here a L-shaped bottom Au electrode (100 nm thick) and an L-shaped top Au electrode (20 nm) are grounded and biased with −0.1 V, respectively. **c**, Cross section illustration of the vOECT from the indicated red dashed line in (**b**). **d**, Optical image of a p-type gDPP-g2T based vOECT array with different top electrode widths but identical bottom electrode. Note, these devices were fabricated by photolithography to accurately pattern the electrode dimensions and locate the semiconductor at the electrode cross points. **e**, Representative transfer characteristics of p-type and n-type (Homo-gDPP) vOECTs for the indicated top electrode width. **f**, Dependence of g_m_ on top electrode width for the p- and n-type vOECTs, where V_D_  = −0.5 V and 0.5 V, respectively. Data are from the average of 8 different devices. **g**, Transfer and g_m_ characteristics of PEDOT:PSS based vOECTs (W = d = 30 µm) and cOECTs (W = 100 µm, L = 10 µm);, V_D_ = −0.1 V. Source data

To our knowledge, there are no examples of truly vertical electrochemical devices in which transistor behaviour is observed throughout the entire semiconductor bulk by using ion-impermeable contacts. Previously reported pioneering organic transistor architectures with vertical source–drain arrangements functioned as OECTs only when using permeable (for example, Ag nanowires) electrodes^22,26,33^, or when operated as electrical double-layer transistors or field-effect transistors. Therefore, bulk ion penetration and redox reactions are not involved, and only a small semiconductor volume under the top contact functions as the charge-carrying channel^34–38^. In contrast, the present approach uses simple thermal evaporation of dense and thick (150 nm) Au electrodes through a shadow mask, in combination with a photopatternable semiconductor layer, to create a structure with excellent ion intercalation. By using a semi-transparent Au top electrode (Extended Data Fig. 6a–c), Supplementary Video 1 demonstrates that the electrochromic switch associated with the redox chemistry encompasses the entire semiconductor area between the top and bottom electrodes and is not confined to the two narrow regions of the semiconductor that are in direct contact with the electrolyte. Evidence for bulk doping is further supported by the following observations: (1) *I*_ON_ and *g*_m_ of the vOECTs with different top electrode widths but identical bottom electrode widths, thus meaning an equal electrolyte–semiconductor interfacial area but a different semiconductor area or mass available for doping or de-doping, increase linearly (Extended Data Fig. 6d–f), illustrating that doping is not limited to the semiconductor–electrolyte interface. (2) Depletion-mode PEDOT:PSS vOECTs exhibit excellent switching behaviour and can be efficiently turned off (Extended Data Fig. 6g), which would be impossible if only interfacial redox chemistry was occurring in the vOECTs. (3) The measured saturation *I*_ON_ values of the present vOECTs would carry unreasonably large electrical current densities (>10^7^ A cm^−^^2^) if the channel were only few nanometres thick, as in typical electrical double-layer transistors. (4) Finally, devices based on very hydrophobic blends, which do not support ion intercalation across the nanoscopic interface, are non-functional (vide supra, Extended Data Fig. 2c).

**Extended Data Fig. 7:**
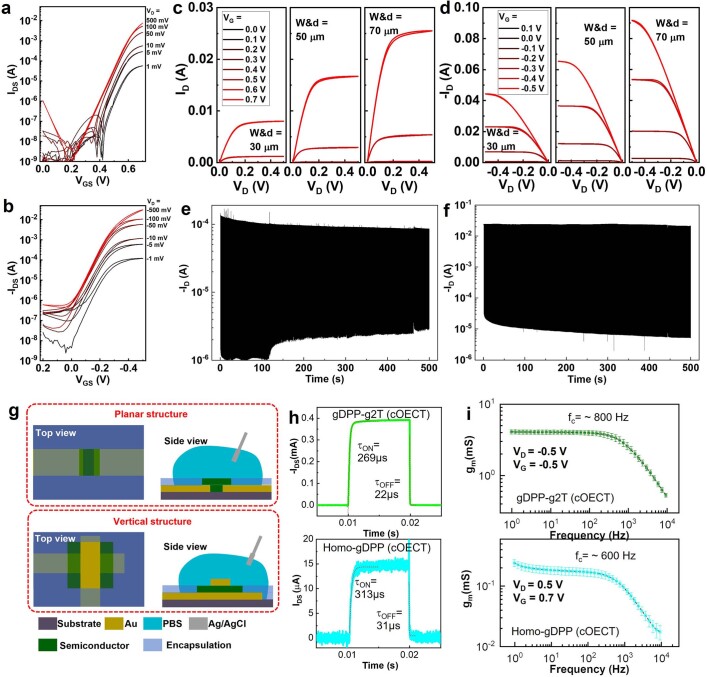
Transfer characteristics, output characteristics, transient properties, and bandwidth characteristics of OECTs. Transfer characteristics of (**a**) n-type **Homo-gDPP:Cin-Cell** and (**b**) p-type **gDPP-g2T:Cin-Cell** vOECTs with V_D_ from ±1 mV to ±500 mV. W = d = 30 µm. Output characteristics of (**c**) n-type **Homo-gDPP:Cin-Cell** and (**d**) p-type **gDPP-g2T:Cin-Cell** vOECTs with W = d = 30, 50, and 70 µm. Cycling stability (cycling frequency of 10 Hz) of PEDOT:PSS based (**e**) cOECT and (**f**) vOECT, where V_D_ = −0.1 V, V_G_ is switching between −0.1 V and 0.7 V with a frequency of 10 Hz. **g**, Illustration of the cOECT and vOECT structures characterized by transient and bandwidth measurements. **h**, Drain current transient responses of cOECTs based on **gDPP-g2T:Cin-Cell**(9:2) and **Homo-gDPP:Cin-Cell**(9:2). (Semiconductor thicknesses are 100 nm, V_G_ is a square pulse from 0.0 to −0.5 V and 0.0 to 0.7 V for p-type and n-type cOECTs, respectively). **i**, Frequency dependent transconductance of cOECTs based on **gDPP-g2T:Cin-Cell** (9:2), and **Homo-gDPP:Cin-Cell** (9:2) (Error bars represent s. d. for N = 6). Cutoff frequencies (f_c_) of ~800 and ~500 Hz were measured for **gDPP-g2T** based cOECT and vOECT, respectively. This result is consistent with the transient response, where both τ_ON_ and τ_OFF_ of the vOECT are greater than those of the corresponding cOECT. In contrast, the f_c_ of Homo-gDPP based vOECT is ~1200 Hz, which is much higher than that in the corresponding cOECT (~ 600 Hz). This reflects the unstable cycling behavior of Homo-gDPP based cOECTs, which degrades the transconductance during bandwidth measurements, especially at high frequencies. Note, in the vertical structure, the cycling stability is greatly enhanced due to the protection of the top electrode which prevents doped polymer film dissolution/delamination, thereby maintaining structural stability. Source data

The present vOECTs also exhibit good transistor behaviour even when operated at a *V*_D_ of only ±0.001 V (Extended Data Fig. 7a,b). Note, especially for the n-type vOECTs, *V*_ON_ shifts from +0.43 to +0.21 V when *V*_D_ is only increased from +0.001 to +0.1 V because of the drain-induced barrier lowering, which is a short channel effect^22^. For n-type cOECTs reported in the literature, and here specifically for Homo-gDPP cOECT control (Extended Data Fig. 4h), the energetic mismatch between the n-type semiconductor LUMO level and the Au electrode work function results in a very high V_ON_ (>+0.4 V), and the limited electrochemical window of the aqueous electrolyte prevents the application of large *V*_G_ biases. This is one of the key limitations of current n-type cOECTs^39^ and it is where drain-induced barrier lowering plays a key role in the n-type vOECT performance enhancement seen here. Common issues of short channel transistors, such as loss of saturation^40^, *V*_T_ roll-off and reduced current modulation^22^, which are equally as important, are absent in the vOECTs (Fig. 2 and Extended Data Fig. 7c,d). This result is possible only if the redox processes modulate the carrier concentration of the entire semiconducting layer^2,41^. The low SS of approximately 60 mV per decade measured for both vOECTs (Fig. 2b,d) provides more convincing proof of the extremely effective gating in the present vertical architecture. Furthermore, unlike cOECTs in which the region with SS approximately 60 mV per decade, if achieved, is narrow (Extended Data Fig. 4g,h), the present vOECTs have a very wide subthreshold region (0.0 ≈ −0.2 V for gDPP-g2T and +0.3 ≈ +0.6 V for Homo-gDPP) with SS near or equalling the approximately 60 mV per decade thermal limit. The wide subthreshold region is particularly useful for applications in which high voltage gain and low power consumption are vital^42,43^.

**Fig. 3:**
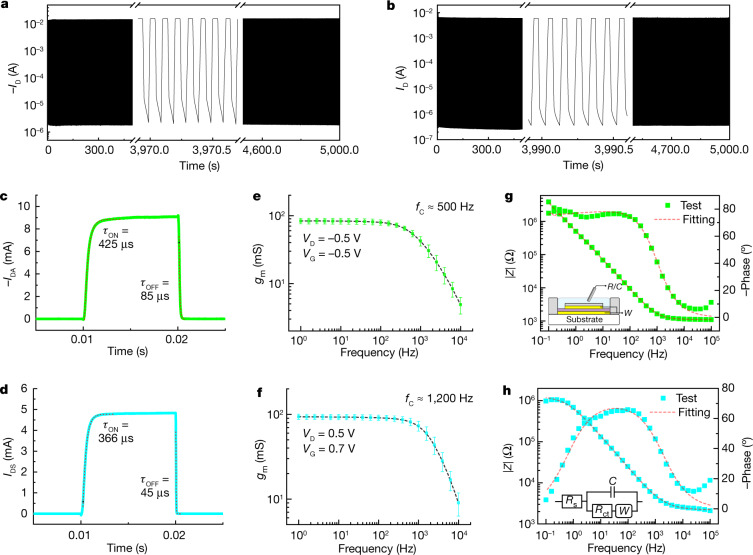
vOECT stability, switching time and frequency-dependent transconductance (bandwidth) characteristics. **a**–**d**, Cycling stability (cycling frequency of 10 Hz) (**a**,**b**) and transient response (**c**,**d**) of p-type gDPP-g2T (**a**,**c**) and n-type Homo-gDPP (**b**,**d**) vOECTs, where *V*_D_ = −0.1 V, *V*_G_ is switching between 0 V and −0.5 V for the p-type vOECT, and *V*_D_ = +0.1 V, *V*_G_ is switching between 0 V and +0.7 V for the n-type vOECT. Note, for both vOECTs, *W* = *d* = 30 µm, *L* *≈* 100 nm. **e**,**f**, Frequency-dependence of the small signal transconductance of p-type gDPP-g2T (**e**) and n-type Homo-gDPP (**f**) vOECTs, where the bias status is indicated in the figure and an additional 10 mV peak-to-peak gate voltage oscillation is applied (error bars represent s.d. for *n* = 6). **g**,**h**, EIS of vertical configuration (|*Z*|) (*W* = *d* = 30 µm, *L* *≈* 100 nm) based on p-type gDPP-g2T:Cin-Cell (**g**) and Homo-gDPP:Cin-Cell (**h**). The insets in **g** and **h** are the EIS measurement setup (**g**) and the equivalent circuit (2R1C behavior) (**h**). Source data

The cycling stability along with the transient response of the OECTs were next assessed. As shown in Fig. 3a,b, for both p- and n-type vOECTs, more than 50,000 stable switching cycles are recorded, which is an order of magnitude higher than for literature values of OECTs, especially for n-type devices^21,44^. Note that the stability of the ubiquitous PEDOT:PSS in depletion-mode vOECTs is also greatly stabilized compared to that in cOECT architectures (Extended Data Fig. 7e,f). Furthermore, the vOECT turn-on transient time (*τ*_ON_) is less than 0.5 ms for both devices (Fig. 3c,d), and is comparable to those of the corresponding precisely patterned cOECTs (Extended Data Fig. 7g,h).

**Extended Data Fig. 8:**
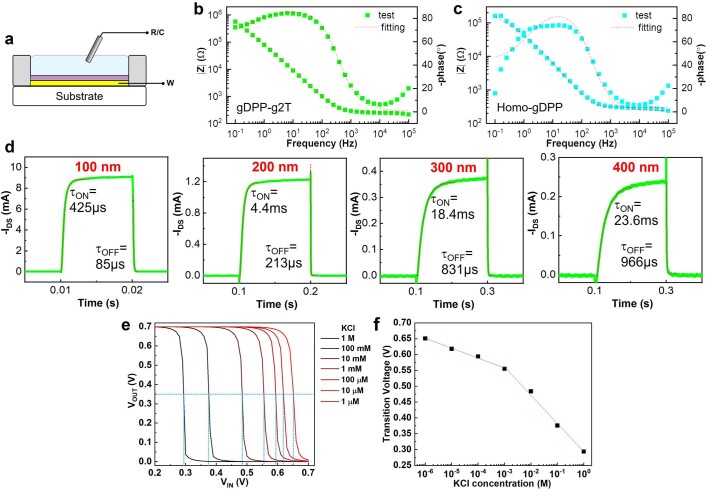
Electrochemical impedance spectroscopy, transient response, and ion sensing using vOECT-based vertically stacked inverters. **a**, EIS measurement setup of typical two-electrode along with the corresponding spectroscopies based on (**b**) **gDPP-g2T:Cin-Cell**(9:2) and (**c**) **Homo-gDPP:Cin-Cell** (9:2). **d**, Drain current transient responses of vOECTs with **gDPP-g2T:Cin-Cell** (9:2) thicknesses of 100 nm, 200 nm, 300 nm, 400 nm. V_G_ is a square pulse between 0.0 and −0.5 V. **e**, Voltage output characteristics of the vOECT-based vertically stacked complementary inverter for ion sensing over a wide concentration range of KCl in water. **f**, Measured transition voltage as a function of KCl concentration. Sensitivities of 31 mV/dec (10^−6^ ~ 10^−3^ M) and 89 mV/dec (10^−3^ ~ 1 M) are obtained with linear approximation (dash line) of the measurements. Source data

To validate the fast switching process and to understand the underlying mechanism, bandwidth and electrochemical impedance spectroscopy (EIS) measurements for both cOECTs and vOECTs were carried out (Fig. 3e,f and Extended Data Figs. 7i and 8). Cut-off frequencies (*f*_c_) of approximately 500 and approximately 1,200 Hz are measured for p- and n-type vOECTs, respectively, which are consistent with the transient responses in Fig. 3c, whereas the EIS based on the vertical configuration also exhibits typical 2R1C behaviour as that in standard two-electrode configurations (Fig. 3g,h and Extended Data Fig. 8a–c). Moreover, vOECTs with different semiconductor film thicknesses were also fabricated and the transient responses were accessed (Extended Data Fig. 8d). As the film thickness (that is, the vOECT channel length) increases from 100 to 400 nm, *τ*_ON_ and *τ*_OFF_ increase from 425 µs and 85 µs (100 nm) to 32.6 ms and 966 µs (400 nm), respectively, showing that the confined electric field in the channel is the key for the fast transient response (Supplementary Video 2). On the basis of these results, it is fast bulk redox instead of interfacial doping near the semiconductor–electrolyte interface that is established, in which the short channel length leads to a strong electric field in the channel that effectively enhances the ion drift velocity, thereby leading to fast doping. Consequently, even in vOECTs for which the ion diffusion length is greater than 15 µm, the vOECT response times are amongst the shortest of the known n-type OECTs and are comparable to current state-of-the-art p-type OECTs, without extensive vOECT electrolyte or electrode patterning optimization^7,8,14,15,21,23–29,45^.

**Fig. 4:**
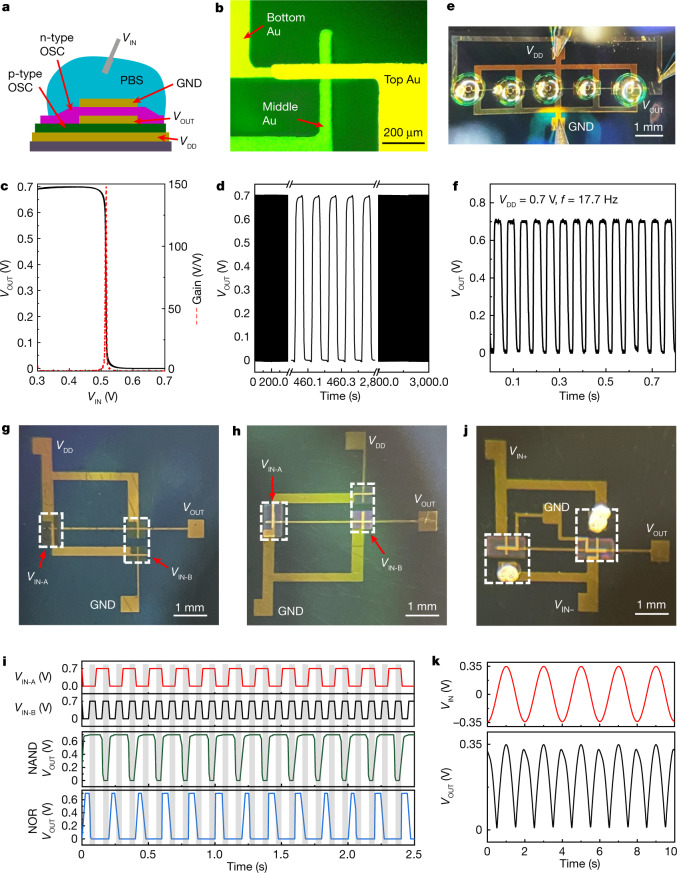
Vertically stacked complementary circuits based on vOECTs. **a**, Illustration of a VSCI based on vOECTs (OSC = organic semiconductor). **b**, Top view of the VSCI, for which the Au electrode locations are indicated. **c**, Voltage output characteristics of the VSCI, along with the voltage gain. **d**, Switching stability of the VSCI with a switching frequency of 10 Hz. **e**,**f**, Photograph of a five-stage ring oscillator (**e**) and the corresponding output characteristics (**f**). **g**–**i**, Photograph of NAND (**g**) and NOR (**h**) circuits, and the corresponding voltage input/output characteristics (**i**). **j**, Photograph of a rectifier and the corresponding output characteristics (**k**). Note, in photographs **g**,**h** and **i**, the electrolyte and Ag/AgCl are omitted to provide a better view of the channel areas. Source data

So far, complementary logic has not been demonstrated for any type of vertical organic transistor architecture, mainly because of immaturity of the fabrication processes^22^. Here vertically stacked complementary inverters (VSCIs) are possible because of the unique vOECT operation mechanism, simple fabrication process and high stability of the present devices. Figure 4a shows a schematic of the VSCI, in which the n-type vOECT is located directly on top of the p-type vOECT. Such three-dimensional geometries enable much higher integration densities that require a 50% smaller footprint per inverter (Fig. 4b). The voltage output characteristics indicate that the VSCI possesses a sharp voltage transition with a gain of up to approximately 150 (driving voltage = +0.7 V, Fig. 4c) and is stable for more than 30,000 switching cycles (Fig. 4d), further corroborating the excellent stability of both the n-type and p-type vOECTs. Thus, the present VSCI can also be used as an effective ion sensor over a wide concentration range (1 µM to approximately 0.1 M of KCl aqueous solution, Extended Data Fig. 8e,f), for which the transition voltage of the inverter can be effectively modulated to near half of voltage drain drain, *V*_DD_/2.

**Extended Data Fig. 9:**
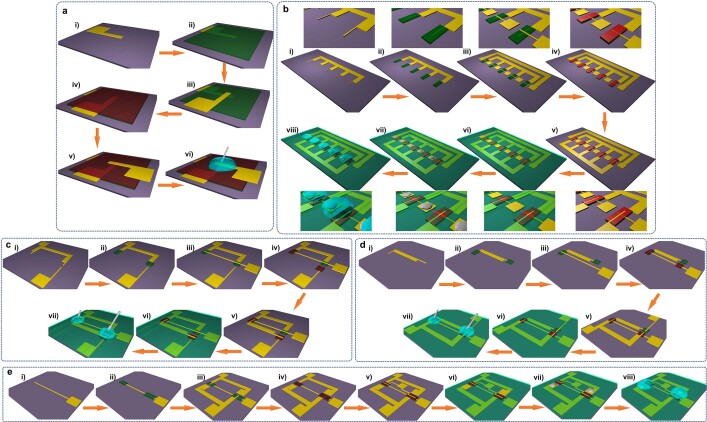
Fabrication process for the Vertically stacked complementary inverter (VSCI) and circuits. **a**, *For the Inverter:* i). Bottom electrodes (V_DD_) evaporation; ii). P-type semiconducting layer fabrication; iii). Middle electrode (V_OUT_) evaporation; iv). N-type semiconducting layer fabrication; v). Top electrodes (GND) evaporation; vi). Application of the PBS electrolyte and Ag/AgCl electrode. **b**, *For the Ring Oscillator* (enlarged plots are also provided)*:* i). Bottom Au electrodes (V_DD_) evaporation; ii). P-type semiconducting layer fabrication; iii). Middle electrodes (V_OUT_) evaporation; iv). N-type semiconducting layer fabrication; v). Top electrodes (GND) evaporation; vi). Cin-Cell encapsulation layer fabrication, where the channel areas and Middle electrode acting as V_OUT_ are left open; vii). Application of the Ag/AgCl paste on top of the Middle electrode V_OUT_ region; viii) Apply the PBS electrolyte. **c**, *For the NAND:* i). Bottom electrodes (V_DD_, and GND) evaporation; ii). P-type semiconducting layer fabrication; iii). Middle electrodes (V_OUT_) evaporation; iv). N-type semiconducting layer fabrication; v). Top electrodes fabrication; vi). Cin-Cell encapsulation layer fabrication, where the channel areas are left open; vii). Apply the PBS electrolyte and Ag/AgCl electrode. **d**, *For the NOR:* i). Bottom electrodes evaporation; ii). P-type semiconducting layer fabrication; iii). Middle electrodes (V_OUT_) evaporation; iv). N-type semiconducting layer fabrication; v). Top electrodes (V_DD_, and GND) evaporation; vi). Cin-Cell encapsulation layer fabrication, where the channel areas are left open; vii). Apply the PBS electrolyte and Ag/AgCl electrode. **e**, *For the rectifier*: i). Bottom Au electrodes (V_OUT_) evaporation; ii). P-type semiconducting layer fabrication; iii). Middle electrodes (V_IN+_, and V_IN-_) evaporation; iv). N-type semiconducting layer fabrication; v). Top electrodes (GND) evaporation; vi). Cin-Cell encapsulation layer fabrication, where the channel areas and the middle electrode acting as V_IN_ are left open; vii). Application of the Ag/AgCl paste on top of the middle electrode V_IN_ region; viii). Apply the PBS electrolyte.

In addition, a five-stage ring oscillator was fabricated based on the present VSCI (Fig. 4e and Extended Data Fig. 9), and the output signal begins to oscillate between 0.0 and +0.7 V at a frequency of 17.7 Hz (*V*_DD_ = +0.7 V, Fig. 4f). This corresponds to a propagation delay of approximately 5.6 ms for each inverter. Finally, NAND and NOR logic gates operating between 0.0 and +0.7 V (Fig. 4g–i and Extended Data Fig. 9) as well as a VSCI-based rectifier (0.35 V amplitude, Fig. 4j,k and Extended Data Fig. 9) were fabricated, demonstrating a versatile library of circuitry elements. Note that previous cOECT-based ring oscillators, NANDs and NORs were fabricated with unipolar p-type cOECTs^17,46–48^, whereas complementary circuits were limited at the preliminary stage of an inverter because of the low performance of the n-type cOECTs^12,14^. Thus, the present vOECTs enable not only VSCIs, which far outperform the corresponding state-of-the-art cOECT inverters^17,49,50^, but they also facilitate the integration of this electrochemical technology into more complex complementary electronics.

In summary, this work reports vOECTs that demonstrate unprecedented performances for both p- and n-type operation modes. The device architecture demonstrated here is enabled by the synthesis of new electro-active and ion-permeable semiconducting polymers and by the interface engineering of electro-active blend layers. The devices are accessible by conventional fabrication processes and provide high fidelity and stable performance characteristics. They open up opportunities for fundamentally new system designs in diverse applications including low-cost diagnostics, brain-machine interfaces, implantable and wearable devices, prosthetics and intelligent soft robotics, for which small effective footprints along with high *g*_m_ and low driving voltage metrics are essential requirements. Moreover, vOECTs offer a new design paradigm for flexible and stretchable complementary devices and related logic circuits.

## Methods

### Materials synthesis

The synthetic route to the new polymers gDPP-g2T and Homo-gDPP is illustrated in Extended Data Fig. 1a. Unless otherwise stated, all reactions were carried out under argon and the solvents were used without any purification. The reagents 2,5,8,11,14-pentaoxahexadecan-16-yl 4-methylbenzenesulfonate (**1**)^51^, 3,6-di(thiophen-2-yl)-2,5-dihydropyrrolo[3,4-c]pyrrole-1,4-dione (**2**)^52^ and 5,5′-bis(trimethyltin)-3,3′-bis(2-(2-(2-methoxyethoxy)ethoxy)ethoxy)-2,2′-bithiophene (**5**)^53^ were synthesized according to previously reported procedures. Hexabutyldistannane (**6**) was purchased from Sigma-Aldrich. The Cin-Cell ploymer was prepared according to our previous publication^54^.

#### Synthesis of 2,5-di(2,5,8,11,14-pentaoxahexadecan-16-yl)-3,6-di(thiophen-2-yl)-2,5-dihydropyr-rolo[3,4-c]pyrrole-1,4-dione (**3**)

Compound **1** (6.00 g, 14.76 mmol), compound **2** (1.84 g, 6.15 mmol), K_2_CO_4_ (4.25 g, 30.75 mmol) and 40 ml of dimethylformamide were added to a 100 ml single-neck round-bottom flask. The reaction mixture was purged with argon for 15 min and was then heated to 150 °C overnight. After cooling to 25 °C, the solvent was removed under reduced pressure. The residue was next dissolved in chloroform and was then washed with water and brine 3 times each. The organic phase was then dried over anhydrous Na_2_SO_4_, filtered and the solvent was removed under vacuum to leave the crude product, which was then purified by silica gel chromatography, eluting with chloroform/methanol (100:1 to 20:1). Compound **3** was obtained as a red solid (1.71 g; yield, 36%). ^1^H NMR (500 MHz, CDCl_3_, Extended Data Fig. 1b): *δ* (ppm) = 8.75 (d, *J* = 3.9 Hz, 2H), 7.64 (d, *J* = 5.0 Hz, 2H), 7.26 (dd, *J* = 5.0 Hz, 3.9 Hz, 2H), 4.26 (t, *J* = 6.3 Hz, 4H), 3.78 (t, *J* = 6.4 Hz, 4H), 3.66–3.51 (m, 32H), 3.36 (s, 6H). ^13^C NMR (126 MHz, CDCl_3_, Extended Data Fig. 1c): *δ* (ppm) = 161.54, 140.44, 134.78, 130.91, 129.68, 128.46, 107.89, 71.94, 70.72, 70.62, 70.60, 70.58, 70.57, 70.52, 68.94, 59.04, 41.88. High-resolution mass spectrometry (HRMS) matrix-assisted laser desorption–ionization (MALDI): calcd for C_36_H_52_N_2_NaO_12_S_2_ (M + Na^+^): 791.2859; found, 791.2851.

#### Synthesis of 3,6-bis(5-bromothiophen-2-yl)-2,5-di(2,5,8,11,14-pentaoxahexadecan-16-yl)-2,5-dihydropyrrolo[3,4-c]pyrrole-1,4-dione (**4**)

Compound **3** (1.00 g, 1.30 mmol) was dissolved in 30 ml of chloroform in a 100 ml single-neck round-bottom flask. The reaction mixture was cooled to 0 °C and *N*-bromosuccinimide  (0.48 g, 2.73 mmol) was added in one portion under argon. The reaction mixture was slowly warmed to room temperature and was stirred overnight in the dark. Water (100 ml) was added and the resutling solution was stirred for 30 min. The organic layer was separated and was dried over anhydrous Na_2_SO_4_, filtered and the solvent was removed under vacuum to leave a residue that was purified by silica gel chromatography with chloroform/methanol (100:1 to 50:1). Compound **4** was obtained as a purple solid (0.86 g, yield 71%). ^1^H NMR (500 MHz, CDCl_3_, Extended Data Fig. 1d): *δ* (ppm) = 8.48 (d, *J* = 4.2 Hz, 2H), 7.20 (d, *J* = 4.2 Hz, 2H), 4.16 (t, *J* = 6.0 Hz, 4H), 3.76 (t, *J* = 6.0 Hz, 4H), 3.66–3.51 (m, 32H), 3.36 (s, 6H). ^13^C NMR (126 MHz, CDCl_3_, Extended Data Fig. 1e): *δ* (ppm) = 161.26, 139.48, 134.86, 131.41, 131.12, 119.35, 107.97, 71.93, 70.76, 70.61, 70.58, 70.56, 70.50, 68.94, 59.03, 42.24, 29.60. HRMS (MALDI): calcd for C_36_H_50_Br_2_N_2_NaO_12_S_2_ (M + Na^+^): 949.1049; found, 949.1044.

#### Synthesis of polymer gDPP-g2T

Compound **4** (92.67 mg, 0.10 mmol), compound **5** (81.62 mg, 0.1 mmol), Pd_2_(dba)_3_ (3.00 mg) and P(*o*-tol)_3_ (7.60 mg) were added to a 10 ml reaction vessel. After the reaction mixture was pump–purged for three cycles with argon, anhydrous toluene (1.5 ml) and dimethylformamide (1.5 ml) were added. The sealed vessel was next heated at 110 °C for 12 h. The polymer was then end-capped with 20 μl of 2-(tributylstannyl)-thiophene and then 50 μl of 2-bromothiophene, with each step being carried out at 110 °C for 1 h. After cooling to room temperature, the mixture was poured into 100 ml of MeOH + 1 ml of concentrated HCl. The resulting precipitate was collected by filtration and then purified by Soxhlet extraction using methanol, acetone, hexane and then chloroform. The chloroform portion was concentrated and then poured into MeOH (approximately 100 ml). The resulting precipitate was collected by vacuum filtration as a black solid (106.51 mg, yield 86%).^1^H NMR (500 MHz, C_2_D_2_Cl_4_, Extended Data Fig. 1f): *δ* (ppm) = 8.73–8.69 (br, 2H), 7.43–6.84 (br, 4H), 4.34–4.25 (br, 8H), 3.94–3.45 (m, 56H), 3.29 (s, 12H). Anal. calcd for [C_58_H_84_N_2_O_20_S_4_]_*n*_: C, 55.40; H, 6.73; N, 2.23. Found: C, 55.41; H, 6.67; N, 2.37.

#### Synthesis of polymer Homo-gDPP

The polymer Homo-gDPP was synthesized by using the same method as used for gDPP-g2T. Compound **4** (199.00 mg, 0.21 mmol), compound **6** (124.57 mg, 0.21 mmol), Pd_2_(dba)_3_ (5.00 mg) and P(*o*-tol)_3_ (13.00 mg) were used as starting materials. The pure polymer was obtained as a black solid (100.00 mg, yield 62%). ^1^H NMR (500 MHz, C_2_D_2_Cl_4_, Extended Data Fig. 1g): *δ* (ppm) = 8.91–8.69 (br, 2H), 7.43–7.22 (br, 2H), 4.25 (br, 4H), 3.78–3.54 (br, 36H), 3.46 (br, 6H). Anal. calcd for [C_36_H_52_N_2_O_12_S_2_]_*n*_: C, 56.23; H, 6.82; N, 3.64. Found: C, 56.26; H, 6.83; N, 3.76.

### Materials characterization

The ^1^H and ^13^C NMR spectra of the intermediates were recorded on a Bruker Ascend 500 MHz spectrometer by using deuterochloroform (CDCl_3_) as the solvent at room temperature. The ^1^H spectra of the polymers were recorded on a Bruker Ascend 500 MHz spectrometer by using dideutero-1,1,2,2-tetrachloroethane (C_2_D_2_Cl_4_) at 100 °C, which was also used to estimate the molecular weight. The purity of the polymers was verified by elemental analysis carried out at Midwest Microlabs Inc.

#### VT–NMR and *M*_n_ estimation by end-group analysis

The solutions for the NMR experiments were prepared by dissolving approximately 5 mg of polymer in 0.7 ml of C_2_D_2_Cl_4_. The solutions were heated at 100 °C for 16 h before the measurements were taken to ensure complete dissolution of the polymer. The measurements were performed on a 400 MHz Bruker Avance III HD Nanobay at 100 °C, and the spectra were referenced to C_2_DHCl_4_ at 5.90 ppm. End-groups were identified based on literature compounds of similar structure^55,56^. Calculation of the *M*_n_ from the end-group analysis is based on equation (1), which is described in the literature^57^.

1 $${n}_{x}=\frac{{a}_{x}{m}_{y}{n}_{y}}{{a}_{y}{m}_{x}}$$

where *a*_*x*_ is the corrected number of repeat unit protons, *m*_*y*_ is the number of end-group protons used for the calculation, *a*_*y*_ is the area of the end-group protons and *m*_*x*_ is he number of repeat unit protons.

For Homo-gDPP: *n*_*x* _= [(10.65)(2)(2)]/[(1)(2)] = 21.3 ≈ 21 and *M*_n _= (21 × 0.76893) = 16.4 kDa.

For gDPP-g2T: *n*_x _= [(16.8)(2)(2)]/[(1)(2)] = 33.6 ≈ 34 and *M*_n _= (34 × 1.16744) = 39.7 kDa.

### OECT and complementary circuit fabrication

#### Semiconductor solution preparation

The gDPP-g2T, Homo-gDPP and Cin-Cell were first dissolved in chloroform at a concentration of 20 mg ml^−1^ and were filtered through a 0.45 µm polyvinylidene difluoride filter. Then, the gDPP-g2T or Homo-gDPP solution was mixed with the Cin-Cell solution in a volume ratio of 9:2 for device fabrication by using the blends. For Pg2T-T, PIBET-AO, PEG-N2200 and BTI2, they were also first dissolved in chloroform at a concentration of 20 mg ml^−1^ and were filtered through a 0.45 µm polyvinylidene difluoride filter, then mixed with the Cin-Cell solution in a volume ratio of 9:2. For PEDOT:PSS (Xi’an Polymer Light Technology Corp.), a solution containing 1 ml of PH1000 (solid content approximately 1.3%, PEDOT content approximately 0.37%), 1 polyethylene glycol dimethacrylate (1.2:1 weight ratio versus PEDOT), 5 wt% of Irgacure 2959 (versus polyethylene glycol dimethacrylate) and 1 wt% of Capstone FS-30 (versus PH1000)^58^ was prepared.

#### Conventional OECT fabrication

A Si wafer with a 300-nm-thick SiO_2_ layer was used as the substrate. It was ultrasonically cleaned, first in an isopropyl alcohol bath for 20 min and then with oxygen plasma for 5 min. The S1813 photoresist was spin-coated at 4,000 rpm for 45 s, followed by annealing at 110 °C for 60 s and was then exposed under a maskless aligner system (MLA150; Heidelberg Instruments), developed in AZ400k (Microchemicals) for 40 s, rinsed with deionized water and blow-dried. Next, 3 nm of Cr and 50 nm of Au were deposited by thermal evaporation and were developed by soaking in acetone for 5 min to remove the S1813. Here, the patterned planar Au source–drain electrodes defined the channel dimension of *W* = 100 μm and *L* = 10 μm. The p- or n-type semiconductor blend solution was then spin-coated at 3,000 rpm for 20 s and was UV cross-linked for 30 s (Inpro Technologies F300S). At last, a droplet (approximately 1–20 µl, based on the channel area) of phosphate buffer solution (PBS, 1×) was applied onto the electrode overlapping area, and an Ag/AgCl electrode was inserted in the droplet acting as the OECT gate electrode. For the well-patterned cOECTs, the fabrication process can be found in the literature^59^, and the devices have patterned semiconductor areas (100 × 20 µm^2^, in which the channel length is 10 µm, the channel width is 100 µm, the channel thickness is 100 nm and the overlap with the source and/or drain is 5 μm on each side) and encapsulated source–drain electrodes.

#### Vertical OECT fabrication

An illustration of the vOECT fabrication process can be also found in Fig. 1a. The vOECTs were also fabricated on a pre-cleaned Si/300 nm SiO_2_ wafer. First, 3 nm of Cr and 150 nm of Au (rate approximately 0.5–2.0 Å s^−1^) were thermally evaporated with a shadow mask as the bottom source electrode. Next, the semiconductor blend solution was spin-coated on the substrate at 3,000 rpm for 20 s. The semiconducting layer was then UV cross-linked for 30 s (Inpro Technologies F300S). Note that the semiconducting layer can be further patterned by developing it in chloroform for 3 s and blow-drying if cross-linked with a photomask. The top drain electrode (150 nm Au) was then thermally evaporated (rate approximately 0.5–2.0 Å s^−1^) with a shadow mask while maintaining the substrate at a temperature of approximately 20 °C with a back water-cooling system. Finally, a droplet (approximately 1–20 µl, based on the channel area) of PBS (1×) was applied on the electrode overlapping area, and an Ag/AgCl electrode was inserted in the droplet acting as the OECT gate electrode. Control devices using the pure semiconductors were fabricated following the same procedure but by using pure polymer solutions and without UV exposure.

#### Complementary inverter fabrication

An illustration of the fabrication process can be found in Extended Data Fig. 9a. For the inverter fabrication, a layer of the opposite type of semiconductor blend was spin-coated (3,000 rpm for 20 s) directly onto the first vOECT (before applying the PBS electrolyte and the Ag/AgCl electrode), and was UV cross-linked for 30 s. Next, the third Au electrode (150 nm) was evaporated with a shadow mask as describe above. Note that the third Au electrode was carefully aligned to overlap with the active area of the bottom vOECT. Finally, a droplet (approximately 1–20 µl, based on the channel area) of PBS (×1) was applied on the electrode overlapping area, and an Ag/AgCl electrode was inserted in the droplet acting as *V*_IN_ of the inverter.

#### Complementary ring oscillator fabrication

An illustration of the fabrication process can be found in Extended Data Fig. 9b. The five-stage ring oscillator was also fabricated on a pre-cleaned Si/300 nm SiO_2_ wafer. First, 3 nm Cr and 150 nm Au were thermally evaporated with a shadow mask as the bottom electrode (*V*_DD_). Next, the p-type gDPP-g2T:Cin-Cell mixture solution was spin-coated on the substrate at 3,000 rpm for 20 s and was cross-linked under UV light for 30 s with a shadow mask. The film was patterned by immersing it in chloroform for 3 s and blow-drying. Next, 150 nm Au were thermally evaporated with a shadow mask as the middle electrode (*V*_OUT_). The n-type Homo-gDPP:Cin-Cell mixture was then spin-coated and photopatterned in the same way as for the p-type polymer blend. Then, 150 nm Au top electrode (ground, GND) was thermally evaporated with a shadow mask. Pure Cin-Cell solution was then spin-coated at 5,000 rpm for 20 s, cross-linked under UV light for 60 s with a shadow mask and developed in chloroform for 3 s, to leave openings for the active channel areas and *V*_OUT_ electrodes. A Ag/AgCl paste (Creative Materials, 125-20) was applied on the *V*_OUT_ electrodes of each inverter and vacuum dried for 30 min. Finally, a drop of PBS electrolyte (approximately 2 µl) was applied on each *V*_OUT_ electrode and its adjacent inverter active channel area.

#### NAND and NOR fabrication

An illustration of the fabrication process can be found in Extended Data Fig. 9c,d. NAND and NOR logic gates were also fabricated on a pre-cleaned Si/300 nm SiO_2_ wafer. First, 3 nm Cr and a 150 nm Au were thermally evaporated with a shadow mask as the bottom electrode. Next, the p-type gDPP-g2T:Cin-Cell mixture solution was spin-coated on the substrate at 3,000 rpm for 20 s and was cross-linked under UV light for 30 s with a shadow mask. The film was patterned by immersing in chloroform for 3 s and blow-dried. Next, 150 nm Au were thermally evaporated with a shadow mask as the middle electrode (*V*_OUT_). The n-type Homo-gDPP:Cin-Cell mixture was then spin-coated and photopatterned in the same way as for the p-type polymer blend but with a different shadow mask. Then, 150 nm Au top electrode was thermally evaporated with a shadow mask. Pure Cin-Cell solution was spin-coated at 5,000 rpm for 20s, cross-linked under UV light for 60 s with a shadow mask and developed in chloroform for 3 s, to leave openings for the active channel areas. Finally, two drops of PBS electrolyte (approximately 2 µl) were applied on each *V*_IN_ area along with two Ag/AgCl electrodes as *V*_IN-A_ and *V*_IN-B_, respectively.

#### Rectifier fabrication

An illustration of the fabrication process can be found in Extended Data Fig. 9e. The rectifier was also fabricated on a pre-cleaned Si/300 nm SiO_2_ wafer. First, 3 nm Cr and 150 nm Au were thermally evaporated with a shadow mask as the bottom electrode (*V*_OUT_). Next, the p-type gDPP-g2T:Cin-Cell mixture solution was spin-coated on the substrate at 3,000 rpm for 20 s and was cross-linked under UV light for 30 s with a shadow mask. The film was patterned by immersing it in chloroform for 3 s and blow-drying. Next, 150 nm Au were thermally evaporated with a shadow mask as middle electrode (*V*_IN+_ and *V*_IN−_). The n-type Homo-gDPP:Cin-Cell mixture was then spin-coated and photopatterned as that of the p-type polymer blend. Then, 150 nm Au top electrode (GND) was thermally evaporated with a shadow mask. Pure Cin-Cell solution was then spin-coated at 5,000 rpm for 20 s, cross-linked under UV light for 60 s with a shadow mask and developed in chloroform for 3 s to leave openings for the active channel areas and *V*_IN_ electrodes. A Ag/AgCl paste (Creative Materials, 125-20) was applied on the *V*_IN_ electrodes and was vacuum dried for 30 min. Finally, two drops of PBS electrolyte (approximately 2 µl) were applied on each *V*_IN_ electrode and its adjacent active channel area.

### Device characterization

#### Transistor measurement

The electrical characterization of the OECTs and inverters was carried with an Agilent B1500A semiconductor parameter analyser in ambient conditions. The voltage sweeping speed was 0.1 V s^−1^ for the OECT measurements. For the transistor and inverter cycling tests, the voltage pulse was generated by a Keysight waveform generator (33500B), whereas the current–voltage variation was monitored with an Agilent B1500A. During the cycling tests, to maintain a relatively stable PBS electrolyte concentration, a PDMS mould was placed on top of the device active area to confine the electrolyte displacement and to slow water evaporation. Transient time measurements were carried out with an FS-Pro (PDA) semiconductor parameter analyser. For the ring oscillator characterization, a constant *V*_DD_ of +0.7 V was applied with an Agilent B1500A, and the *V*_OUT_ was monitored by an oscilloscope (Tektronix, TDS 2014). For NAND and NOR characterization, square pulses (from 0.0 to ±0.7 V) with a frequency of 5 Hz and 10 Hz were applied as *V*_IN-A_ and *V*_IN-B_, respectively, by a Keysight waveform generator (33500B), and *V*_OUT_ was monitored by an Agilent B1500A. For rectifier characterization, two sinusoidal *V*_IN_ (*V*_IN+_ and *V*_IN−_ have a phase difference of 180°) with an amplitude of 0.35 V were generated by a Keysight waveform generator (33500B), and the *V*_OUT_ was monitored by an Agilent B1500A. All measurements were carried out in ambient conditions.

#### EIS measurements

All measurements were conducted by using a PalmSens4 potentiostat (PalmSens) with an Ag/AgCl pellet (Warner Instruments) as the reference and counter electrode, and a gold electrode coated with active materials as the working electrode. For measurements on the vertical structure, details can be found in Extended Data Fig. 7, which were performed in PBS (1×) electrolyte with a direct current offset as 0.5 V (for the p-type material) and −0.7 V (for the n-type material), superimposed by a 10 mV alternating current (a.c.) oscillation. The frequency of the a.c. oscillation ranges from 0.1 to 10^5 ^Hz.

#### Bandwidth measurements

Bandwidth measurements were conducted by accessing the *g*_m_ of the OECT as a function of the frequency of the gate voltage oscillation. The National Instruments (NI) SMU unit (NI PXIe-4143) was used for sourcing and measuring the drain–source voltage and current, as well as the gate current. The gate voltage was applied by using the data acquisition (DAQ) card from NI (NI PXIe-6363)] and was measured with a NI BNC-2110. During the measurement, *V*_DS_ is equal to −0.5 V (p-type) and 0.5 V(n-type), whereas *V*_G_ is equal to −0.5 V (p-type) and 0.7 V (n-type), superimposed by a 10 mV a.c. oscillation. The frequency of the a.c. oscillation ranges from 1 to 10^4 ^Hz. All measurements were automated by using a custom LabVIEW programme (NI) and the data were processed by using the MATLAB software (Mathworks).

### Semiconductor film characterization

SEM characterizations were carried out on a Hitachi SU8030 FE-SEM. AFM characterizations were acquired with a Bruker ICON System. GIWAXS measurements were performed at Beamline 8-ID-E1 at the Advanced Photon Source (APS) at Argonne National Laboratory. Samples were irradiated with a 10.9 keV X-ray beam at an incidence angle 0.125° to 0.135° in a vacuum for two summed exposures of 2.5 s (totalling 5 s of exposure), and scattered X-rays were recorded by a Pilatus 1 M detector located 228.16 mm from the sample at two different heights.

### Discussion of the calculated mobilities of cOECT and vOECTs

The carrier mobilities of both the gDPP-g2T:Cin-Cell and Homo-gDPP:Cin-Cell were measured in vertical and planar OECTs^59^. For the planar or conventional architecture, the carrier mobility of the p-type cOECT is found to be 1.69 ± 0.19 cm^2 ^V^−1^ s^−1^, which is comparable to other high-performance p-type OECTs. The n-type cOECT exhibits a high mobility of 0.13 ± 0.03 cm^2 ^V^−1 ^s^−1^, which is among the highest reported up until now. However, for the vertical devices, the calculated carrier mobilities are much lower, (3.33 ± 0.27) × 10^−3^ cm^2 ^V^−1 ^s^−1^ and (3.06 ± 0.61) × 10^−3^ cm^2 ^V^−1 ^s^−1^ for the p-type and n-type vOECTs, respectively.

The much lower and similar carrier mobilities in the vOECTs probably originate from the large series resistance from the source–drain electrodes and, to a lesser extent, the non-optimal polymer morphology of the spin-coated films, which typically enhances in-plane rather than out-of-plane organic semiconductor charge transport. Because the measured channel resistance in the vertical structure in the on-state is less than 10 Ω, series resistances originating at the electrode–semiconductor interface and within the electrode contact will reduce the measured drain current notably^16^. Overall, these observations indicate that further optimization of charge injection and connecting-line conductivity, and the use of organic semiconductors that favour vertical charge transport as in those for organic photovoltaics, will probably enhance the current densities even further. Nevertheless, to properly evaluate the true carrier mobilities for the unconventional vertical architecture reported here, additional modelling and simulation efforts are required. This would be of great interest to the entire community.

## Online content

Any methods, additional references, Nature Portfolio reporting summaries, source data, extended data, supplementary information, acknowledgements, peer review information; details of author contributions and competing interests; and statements of data and code availability are available at 10.1038/s41586-022-05592-2.

### Supplementary information

Supplementary Video 1 Top view video of a p-type gDPP-g2T:Cin-Cell vOECT with a large electrode overlapping area (approximately 2 × 0.5 mm^2^). The electrochromic process associated with the redox chemistry of the semiconducting layer can be observed in this video. Here the bottom Au electrode (100 nm) and the top Au electrode (20 nm) are biased with 0 and −0.1 V, respectively. The voltage bias applied on Ag/AgCl electrode is indicated in the video.

Supplementary Video 2 Top view video of an electrolyte capacitor based on a p-type gDPP-g2T:Cin Cell film. The voltage bias applied on the Ag/AgCl electrode is indicated in the video. Note that the ellipsoid visible in the middle of the video is the reflection of the microscope light illuminating the device during the recording. When *V*_G_ is switched from 0 V to −0.7 V, the polymer film located in direct contact with the Au electrode oxidizes immediately (in 100 ms in a 2 × 0.5 mm^2^ area). This reflects the high electric field (*E*) near Au increasing the ion drift velocity, *s* (*s* = *μ*_i_*E*, where *μ*_i_ = ion mobility), thus promoting faster doping. Next, as the charging process continues, the entire electrolyte area covered semiconductor (approximately 6 × 2.5 mm^2^) oxidizes slowly (approximately 2 s), starting from the semiconductor near the Au electrode. Similarly, the reduction also begins from the semiconductor in direct contact with the Au electrode, followed by the portion near the edges. Therefore, since the entire vOECT channel layer is in direct contact with the Au electrode, the redox process is intrinsically fast.

### Source data

Source Data Fig. 2

Source Data Fig. 3

Source Data Fig. 4

Source Data Extended Data Fig. 1

Source Data Extended Data Fig. 2

Source Data Extended Data Fig. 3

Source Data Extended Data Fig. 4

Source Data Extended Data Fig. 5

Source Data Extended Data Fig. 6

Source Data Extended Data Fig. 7

Source Data Extended Data Fig. 8

## Figures and Tables

**Extended Data Table 1:**
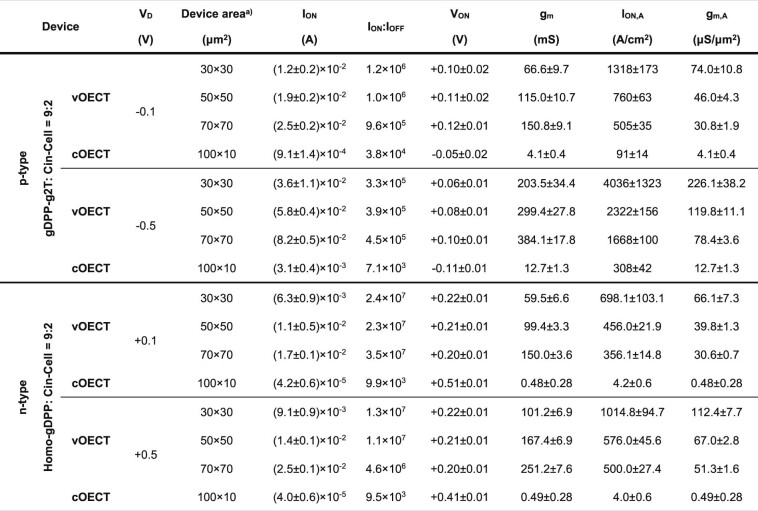
Summary of OECT performance metrics

^a)^ For vOECT, the channel area is W×d; for cOECT, the area is W×L. ^b)^ The standard deviation of the OECT metrics is from the performance of at least 10 devices. ^c)^ Here the volume-normalized g_m_ is not reported, since it is a parameter only suitable for evaluating the performance of a material in the same architecture. Importantly, for real-world applications, a transistor footprint (area-normalized) g_m_ and current are far more relevant when fabricating circuits, as they determine how dense the OECT integration can be while retaining high performance. Thus, the goal in most transistor circuitry fabrication is not to achieve high geometry-normalized g_m_, but high g_m_/current in limited areas, which are the I_ON,A_ and g_m,A_ reported here. Similarly, the calculated mobilities of cOECTs and vOECTs are discussed in Methods.

## Data Availability

Source data are provided with this paper. Additional data related to this work are available from the corresponding authors upon request.
